# Currently Available Monitoring and Surveillance Systems for *Taenia* spp., *Echinococcus* spp., *Schistosoma* spp., and Soil-Transmitted Helminths at the Control/Elimination Stage: A Systematic Review

**DOI:** 10.3390/pathogens9010047

**Published:** 2020-01-06

**Authors:** Ganna Saelens, Sarah Gabriël

**Affiliations:** Department of Veterinary Public Health and Food Safety, Faculty of Veterinary Medicine, Ghent University, Merelbeke B-9820, Belgium

**Keywords:** neglected tropical diseases, monitoring and surveillance, helminth zoonoses, *Taenia* spp., *Echinococcus* spp., *Schistosoma* spp., soil-transmitted helminths

## Abstract

An increasing global focus on neglected tropical diseases (NTDs) has resulted in the set up of numerous control and elimination activities worldwide. This is partly true for *Taenia solium* taeniasis/cysticercosis, the most important foodborne parasitic infection. Despite substantial progress, adequate monitoring and surveillance (M&S) are required to sustain a status of control/elimination. This is often lacking, especially for *T. solium*. Therefore, the objective was to conduct a systematic literature review of the currently available M&S systems at the control/elimination stage of the four top-ranked helminth NTDs. Specifically, *Taenia* spp., *Echinococcus* spp., *Schistosoma* spp., and soil-transmitted helminths (STHs) were considered to determine if there are any similarities between their M&S systems and whether certain approaches can be adopted from each other. The systematic review demonstrated that rigorous M&S systems have been designed for the control/elimination stage of both STHs and schistosomiasis, particularly in China. On the other hand, a concept of M&S for *Taenia* spp. and *Echinococcus* spp. has not been fully developed yet, due to a lack of epidemiological data and the fact that many endemic countries are far away from reaching control/elimination. Moreover, accurate diagnostic tools for all four diseases are still imperfect, which complicates proper M&S. Finally, there is an urgent need to develop and harmonize/standardize M&S activities in order to reliably determine and compare the epidemiological situation worldwide.

## 1. Introduction

Neglected tropical diseases (NTDs) currently pose significant health risks to more than one billion people worldwide and cause substantial economic losses in the health and food sectors. They are traditionally believed to affect populations of developing countries with limited adequate sanitation and close contact with livestock; however, their frequencies are rising in developed, urban areas due to the influx of immigrants and increase in travel. As a result, tropical diseases have been increasingly subjected to integrated control activities in the past century, particularly in developing countries [1]. *Taenia solium*, for instance, is an NTD ranked first on the global scale of parasitic foodborne diseases with an estimated 2.5 million people all over the world carrying this pork tapeworm and 50 million people being infected with *T. solium* cysticerci [2]. Although substantial progress has been made in combating this helminthic zoonosis, it is often difficult to sustain control or elimination without adequate monitoring and surveillance (M&S) [3]. The M&S systems, although pivotal, are usually lacking in the case of *T. solium* [4]. Therefore, a first objective was to conduct a systematic review on the currently available M&S systems at the control/elimination stage of *T. solium*. Given the deficiency of these systems for *T. solium*, the review also covered the beef tapeworm *Taenia saginata* due to its close taxonomic relationship with *T. solium.* Although *T. saginata* infection is not considered an NTD, it was included to determine if there are any similarities between their M&S systems and whether certain approaches can be adopted from each other. Finally, given that the outcome for both the *Taenia* spp. was low, three other top-ranked helminthic NTDs (caused by *Echinococcus* spp., *Schistosoma* spp., and soil-transmitted helminths (STHs)) were added to look for any parallels in their M&S systems.

Before comparing the different M&S systems of *Taenia* spp., *Echinococcus* spp., *Schistosoma* spp., and STHs, it is essential to define and distinguish some epidemiological concepts for M&S data collection and management. Disease monitoring refers to the ongoing efforts of collecting, analyzing, and distributing all possible data about health, disease, and their determinants in a given population over a defined period of time [5]. Monitoring a control program, on the other hand, refers to the process of collecting and analyzing information about the effect of an intervention on the health status of a given population or environment. In other words, it ensures that the implemented interventions are effective and efficient, and is particularly important at the control stage of a disease. Monitoring is often paired with evaluation; the process that attempts to determine as systematically and objectively as possible the relevance, effectiveness, and impact of the intervention programs [6]. Although surveillance consists of a set of monitoring systems, both terms should not be confused. Specifically, disease surveillance is a more active system that only collects a minimal set of essential, spatiotemporal data designed in a way that some form of directed action can be taken whenever the data passes a certain threshold value. For instance, an effective surveillance system at the elimination stage of a disease entails the rapid detection of remaining or re-emerging pockets of transmission, the detection of possible drug resistance, the identification of low transmission/risk areas, and the comprehension of patterns in disease epidemiology (e.g., shifts in age and seasonality) [7]. In sum, when reaching the final crucial stage of transition from general disease control to effective elimination, a shift from comprehensive monitoring and evaluation for measuring morbidity and mortality toward a rigorous surveillance is required. In other words, the choice of approach depends on the stage of control/elimination achieved [2].

Given the lack of implemented M&S systems for *T. solium* and their crucial role in the sustainment of control/elimination after the implementation of control interventions, the general objective of this systematic literature review was to collect and summarize records from English scientific literature on the currently available M&S systems for *Taenia* spp., *Echinococcus* spp., *Schistosoma* spp., and STHs at the control/elimination stage. A first objective was to identify and collect data by using a combination of search terms and key elements in five search engines. A second objective was to retrieve the records relevant to this systematic review through three screening phases. A final objective was to extract and summarize specific information from the selected records in order to obtain a full picture on the existing M&S systems at the control/elimination stage. Additionally, existing gaps in these systems were identified in order to determine potential needs for improvement. In this way, this review may contribute to the establishment of rigorous M&S systems for future NTDs interventions, especially in the case of *T. solium*.

## 2. Methods

### 2.1. Review Question, Search Literature, and Literature Sources

‘What are the currently available M&S systems at the control/elimination stage of four selected NTDs caused by the following parasites: *Taenia* spp., *Schistosoma* spp., *Echinococcus* spp., and STHs?’

This systematic literature review was conducted by implementing the PRISMA (Preferred Reporting Items for Systematic review and Meta-Analysis) guidelines [8]. A completed PRISMA checklist is included in the Supplementary Material to this article. Five search engines (PubMed, Web of Science, ScienceDirect, Google Scholar, and Google Search) were used to identify articles and five domains were included in this search, each with their own key elements: *Taenia* spp. (*Taenia solium* or *Taenia saginata* or tapeworm or taeniosis or taeniasis or cysticercosis), *Schistosoma* spp. (schistosome or schistosomiasis or bilharzia or bilharziasis), *Echinococcus* spp. (or echinococcosis), STHs (soil-transmitted helminths or soil-transmitted helminthiasis), and surveillance and monitoring (survey or surveil or monitor). Search terms and key elements were combined using the Boolean operators (AND, OR, NOT) resulting in the following search algorithms:

- (*Taenia solium* OR *Taenia saginata* OR tapeworm OR taeniosis OR taeniasis OR cysticercosis) AND (surveillance OR surveil OR survey OR monitoring OR monitor).
- (*Schistosoma* OR schistosome OR bilharzia OR bilharziasis) AND (surveillance OR surveil OR survey OR monitoring OR monitor).
- (*Echinococcus* OR echinococcosis) AND (surveillance OR surveil OR survey OR monitoring OR monitor).
- (STHs OR soil-transmitted helminths OR soil-transmitted helminthiasis) AND (surveillance OR surveil OR survey OR monitoring OR monitor).

### 2.2. Study Selection

The records included for this literature review were retrieved through three screening phases. The first screening removed duplicates (titles) after merging the results obtained from the different literature sources. A second screening phase evaluated titles and abstracts regarding the relevance to the study question, while the last phase was applied to the full texts. Four exclusion criteria were applied on the second screening phase: (i) articles written before 2004, (ii) articles not available in English, (iii) articles not containing at least one of the five domains in their titles, and (iv) articles with no available full text. The main reasons for exclusion in the last screening phase were mentioning the need for M&S and the description of M&S in general rather than at the control/elimination stage.

The final selected articles were first categorized by parasite (*Schistosoma* spp., STHs, *Taenia* spp., *Echinococcus* spp., and combinations). Next, records from each category were ordered by topic: M&S field studies, theory about M&S, purely diagnostic matter, and application of spatial technology. The last two topics are briefly discussed at the end of this review, considering the major role of diagnostic tools and spatial technology with regard to M&S.

From the final selected articles, the following data were extracted: name of parasite discussed, the timeframe, the country or region, the method of M&S, the existing gaps in the systems, and potential solutions (if any).

## 3. Results/Discussion

**Figure 1:**
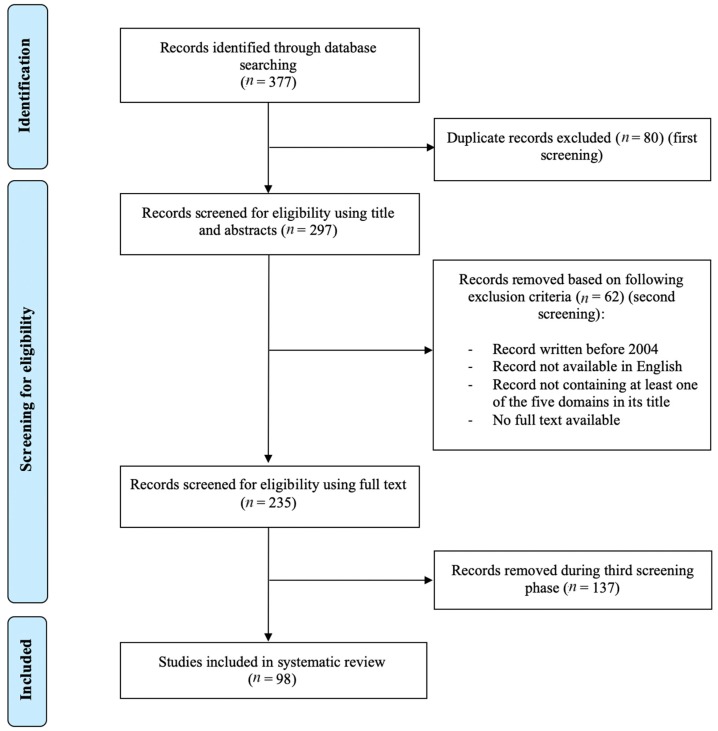
PRISMA (Preferred Reporting Items for Systematic review and Meta-Analysis) flowchart diagram of the record selection process.

The literature search identified a total of 377 records (149 by PubMed, 108 by Web of Science, 73 by ScienceDirect, 43 by Google Scholar, and 4 by Google Search). Eighty duplicates were removed after the first screening and 62 records were removed after the second screening. A substantial number (*n* = 137) of the articles thus obtained were subsequently removed as they did not discuss M&S at the control/elimination stage, which could only be detected during the last screening of the full texts. A total of 98 articles were retained for this systematic literature review (Figure 1). The majority of the remaining records discussed post-control/elimination M&S with regard to *Schistosoma* spp. and STHs (30 and 24 records, respectively), while only 10 and 11 records discussed post-control/elimination M&S with regard to *Echinococcus* spp. and *Taenia* spp., respectively. Finally, 23 records discussed post-control/elimination M&S with regard to a combination of two or more of these NTDs.

### 3.1. Schistosoma *spp*.

#### 3.1.1. Introduction

Schistosomiasis is a parasitic, zoonotic disease caused by infection with the trematode genus *Schistosoma*. Three species are mainly accountable for public health problems worldwide: *Schistosoma mansoni, Schistosoma haematobium*, and *Schistosoma japonicum*. Infection results from contact with water contaminated with free-swimming cercariae that are shed from intermediate snail hosts. Subsequently, the rapid onset of acute schistosomiasis with symptoms, such as high fever, myalgia and eosinophilia, is triggered by the migration of the parasite in the body. Furthermore, a chronic schistosome infection may lead to long-term complications, such as portal hypertension, anemia, bladder cancer, and kidney failure. Additionally, various animals, such as cattle, dogs, cats, pigs, horses, and rodents, may serve as reservoir hosts for *S. japonicum* [9].

The disease represents a worldwide health problem with approximately 240 million people being infected and nearly 800 million people believed to be at risk. Nevertheless, substantial progress in reducing the burden of schistosomiasis in many countries has been achieved due to the extensive implementation of large-scale control interventions. A pioneer in the control and elimination of *S. japonicum* is the People’s Republic of China (PRC) with its strong political commitment and efficient control efforts. With a variety of approaches developed over the past two decades, such as chemotherapy, snail control, health education, improved sanitation, and access to safe water, the PRC aims to achieve nationwide transmission interruption by 2020 and disease elimination by 2025 [10]. In fact, due to the implementation of these control measurements in a national schistosomiasis prevention and control plan, transmission control and interruption (i.e., elimination) have already been achieved in 135 and 313 counties, respectively. In order to sustain these accomplishments, an overall surveillance and consolidation work has been unfolded in the PRC. Therefore, the outcome of the literature search included many records regarding successful M&S systems for *S. japonicum* designed by the PRC. Finally, two records were on monitoring systems in Egypt, a country that is currently on track for complete elimination of *S. mansoni* schistosomiasis.

The scope and design of M&S systems for *Schistosoma* spp. depend on whether the stage of transmission control or transmission interruption has been reached so far. In case of control status, the emphasis is on the monitoring of local residents and livestock (i.e., identification and treatment of patients and livestock with schistosomiasis), suspected snail infestations, and factors associated with schistosomiasis. On the other hand, in case of transmission interruption, the major surveillance tasks are the detection of snails and the follow-up of risk factors and imported infection sources. Furthermore, to remain successful in the long-term, this surveillance needs to be followed by an appropriate and immediate response system at any sign of resurgence of transmission in areas where disease elimination had been declared (e.g., treatment of positive cases, examination of contact persons, and focal mollusciciding of identified freshwater bodies) [11,12].

In sum, the M&S indicators for schistosomiasis include the following: schistosomiasis cases (acute, chronic, and advanced), intermediate snail hosts (their habitat, density, and infection rate), and related epidemic factors (water level, rainfall, temperature, lifestyle of the population, and implementation of control measures). An additional indicator for *S. japonicum* is livestock as a reservoir host (i.e., mainly cattle, but also sheep, pigs, and horses). A thorough and consistent M&S system should consequently take into account each of these indicators and should be robust under spatial and temporal fluctuations. Nevertheless, some records describe a system that solely includes one or two of these indicators. Therefore, this review will first discuss each surveillance indicator separately and finally end with the records describing a combination of these indicators (Table 1).

#### 3.1.2. Surveillance of the Snail Intermediate Host

**Figure 2:**
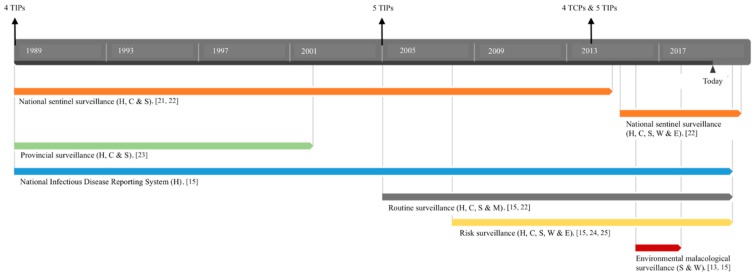
Timeline of different, parallel schistosomiasis surveillance systems in the People’s Republic of China with each of their surveillance indicators. TIP, transmission-interrupted province; TCP, transmission-controlled province; H, human surveillance; C, cattle surveillance; S, snail surveillance; M, other mammalian intermediate hosts surveillance (e.g., dogs); W, water bodies surveillance; E, environmental surveillance.

Considering that the transmission of schistosomiasis is particularly determined by the existence and geographic distribution of its intermediate snail host, it is crucial to determine and pursue the spatial distribution, habitat, and infection rate of these snails. Many ecological and environmental factors, such as light, water, temperature, and aquatic vegetation, may influence this distribution. Furthermore, it must be noted that each *Schistosoma* species has its own snail species as the intermediate host. In order to know if and where a suitable intermediate host is present, snail sampling at several study sites across the country should be conducted. An illustration of this is the malacological survey of *Biomphalaria straminea*, the intermediate host of *S. mansoni*, to determine its dispersal in the Guangdong province (China), where schistosomiasis has been eliminated (Figure 2). At 186 sites that were selected based on the presence of aquatic habitats and the existence of the snail species according to previous studies, snails in a radius of 2 m were captured, labeled, and anatomically identified in a laboratory. Furthermore, water and sediment samples in addition to landscape and climatic data from the study sites were collected and analyzed in order to estimate the relationship between the presence of *B. straminea* and environmental and physicochemical variables. This survey revealed the wide distribution of this snail and a correlation with water temperature and physicochemical properties of sediments (i.e., sediment zinc and water temperature were higher in places with *B. straminea*), meaning that the monitoring of the dispersal and infection rate of this snail must be continued [13]. Further detailed descriptions of malacological surveys, incorporated in an integrated surveillance system, are discussed below (Section 3.1.6).

#### 3.1.3. Monitoring and Surveillance for Human Schistosomiasis Cases

As indicated above, a surveillance system of the human population needs to be put in place when achieving the criteria for transmission interruption in order to prevent re-emergence. A first example of such a system is the one established in the Zhejiang province (China), a formerly highly endemic area for *S. japonicum* where elimination was achieved in 1995 after 40 years of intensive control measures. Here, villagers at risk (i.e., people living in areas still infested with the *Oncomelania* snail host, children under 14 years, people returning from schistosome-endemic areas, and boat dwellers) and suspected patients (i.e., people with symptoms of hepatic fibrosis, portal hypertension, and splenomegaly) were screened for *S. japonicum* using immunological diagnostic tests. When these were found to be positive, fecal examination with the Kato-Katz (KK) technique was followed. In case *S. japonicum* eggs were detected in the fecal samples, the patient was treated with praziquantel (PZQ) and regularly re-examined until complete cure (i.e., negative fecal examination). Additionally, advanced cases of schistosomiasis were continuously subjected to supervision and surveillance (i.e., liver function tests, laboratory examination, and health check-ups). Seven years post-transmission and after 621,431 serological screenings, the system only identified two patients between 1995 and 2002 with both the immunological tests and fecal examination. Although this had proved to be a very effective and recommended surveillance system to be pursued in the years to come, one limitation must be mentioned. Namely, out of all the 621,431 serological tests, an overall positive rate of 2.9% was found, while only two of them were positive when subjected to fecal examination. This demonstrates that the immunological processes in the host’s body do not halt when the parasite is eliminated and therefore, might give false positive results. In conclusion, this surveillance system has the potential to be cost-effective; however, it warrants further research into more sensitive and specific diagnostic tools [14]. Further detailed description and incorporation of human surveillance efforts in an integrated surveillance system will be discussed below (Section 3.1.6). 

A second example of M&S of humans is the establishment of the National Infectious Disease Reporting System (NIDRS) by the Centers of Disease Control and Prevention (CDC) in 1950. This system covers at present 39 infectious diseases, among them schistosomiasis, and its use was mandated by law in 1989 in the PRC (Figure 2). In this country, all levels of the staff of medical institutions are required to report both acute and chronic cases of schistosomiasis diagnosed in hospitals to the NIDRS. Contents of this report system include name, age, gender, address, contact information, occupation, history of treatment, time of diagnosis, diagnostic results, clinical symptoms, characteristics of infection site, and case classification. This reporting system not only helps the development of appropriate prevention and control programs, but is also crucial to reveal national patterns of disease transmission. Nevertheless, disparities in clinical resources (e.g., diagnostic capabilities, presence of computers, and network coverage) may result in disproportional reporting across the country [15].

A similar system was established in Europe in 1999. Due to an increasing number of immigrants, in addition to changing travel patterns, the European Network on Imported Infectious Disease Surveillance (TropNetEurop) was founded in order to facilitate a better understanding of the epidemiology of imported schistosomiasis and to improve pretravel advice. Every patient with a diagnosis of schistosomiasis is reported to TropNetEurop and further classified according to patient characteristics (immigrants, refugees, business travelers, etc.) and reason for travel. Next, individual data are stored in a database which offers a unique tool to monitor changes and to function as an early warning system. Nevertheless, schistosomiasis is a non-notifiable disease in Europe, making data entry self-selected. This inevitably results in underreporting and therefore, this network cannot provide representative data throughout Europe [16].

#### 3.1.4. Monitoring and Surveillance of Livestock

In working from control toward the elimination of zoonotic schistosomiasis, surveillance of the animal reservoir (i.e., cattle, water buffalo, goats, sheep, pigs, and dogs) should also be included in elimination guidelines, considering that they often share the same water points. As such, cattle can infect snails present in water points used by humans, where people can then get infected. As a response, selective treatment or isolation of the infected animals has been proposed as a practical approach to prevent further contamination of the environment [17]; however, these treated animals will most likely just drink from the infected water again and/or work in infected rice fields. Therefore, an M&S system solely including livestock is not expected to be very efficient. Specifically, it should be incorporated in an integrated surveillance system which will be discussed below (Section 3.1.6).

#### 3.1.5. Monitoring and Surveillance of *Schistosoma*-Infected Water

Another important surveillance measure is to detect water sources contaminated with miracidia and cercariae. For the detection of *S. japonicum*-infested water, an intelligent device with sentinel mice was developed. Sentinel mice are left in cages on the water surface in a way that their abdomen and tail are exposed to the water for four hours a day, two days in a month, for four months. To allow maturation of any infecting schistosome, 35 days after the last exposure, the mice are killed and dissected for the presence of eggs in the liver or worms in the portal and mesenteric veins. Although, this device has played a critical role in the surveillance of schistosomiasis in China, it is rather time-consuming and impractical on a large-scale, and poses obvious animal welfare questions. For instance, a study mentioned a 10% loss of the mice due to cages floating away and mice accidentally drowning or suffocating during feeding [18]. Furthermore, the morphology of cercariae from different species is nearly identical which makes determination at the species level without molecular tools extremely challenging, if not impossible [19]. As a result, McManus and colleagues (2018) suggested in 2018 the testing of water samples for environmental schistosome DNA to identify schistosomiasis transmission areas. A study on such a surveillance system had already been carried out in Madagascar in 2016 where an environmental DNA (eDNA) qPCR targeting one specific fragment of the *cox1* (cytochrome c oxidase subunit 1) mitochondrial gene from *S. mansoni* was developed. The results of this study indicated that this tool successfully detects *S. mansoni*-contaminated water. Furthermore, the greatest benefit of the eDNA method is the fact that it enables the collection of data from several sites within a short period of time due to the ease of collecting samples. Nevertheless, further assessment of its cost-effectiveness, potential adaptation to other *Schistosoma* species, and verification in other areas is necessary in the future. In addition, ways to differentiate between the presence of schistosome DNA and the presence of actual viable cercariae will have to be developed. Once established, the environmental qPCR could serve as a promising and feasible M&S tool [20].

#### 3.1.6. Integrated Monitoring and Surveillance Systems for Schistosomiasis

The first integrated system of surveillance described here is a national sentinel surveillance system initiated in 1989 in China when elimination had already been reached in four provinces (Figure 2). It consisted of 20 sentinel sites dispersed over the entire country and representing the different eco-epidemiological settings. The prevalence and intensity of human infection were monitored over a 5-year period with repeated annual cross-sectional surveys and a response rate of 90%. On the other hand, the infection rate in cattle and snails was monitored longitudinally. The surveillance of human population concerned residents between 5 and 65 years of age and was executed in two different ways. The first method in 13 sites screened the people with serodiagnostic techniques (i.e., indirect hemagglutination assay (IHA) or enzyme-linked immunosorbent assay (ELISA)), while the presence of eggs in feces (i.e., the KK technique) confirmed a positive serological test. The second method in the seven remaining sites was based only on the KK technique and results were read by two laboratory technologists. In both the settings, acute and advanced cases of schistosomiasis were regularly examined and interviewed with a standardized questionnaire to reveal the spatial distribution and (environmental) risk factors. Additionally, any individual that tested positive on the KK technique was administered a single oral dose of PZQ. Subsequently, the surveillance of cattle in each of the sentinel sites occurred using the stool hatching method, while snails were collected during spring and autumn for microscopic examination and recording of various indices (i.e., habitat, density of living snails, density of infected snails, and snail infection rate). Chemotherapy with PZQ was implemented in infected livestock whenever cattle positive for schistosomiasis had been detected, and focal mollusciciding was applied in case snails had been found. At the same time, all the people and cattle in these areas were screened (and treated when positive) for schistosomiasis in case infected snails had been detected. Results from this system (between 2000 and 2003) confirmed that schistosomiasis had been successfully controlled in large areas of the PRC; however, infected snails were found in areas where transmission control had been declared [21]. As a result, China continued the ongoing efforts of snail monitoring and control, especially in areas deemed free from schistosomiasis. In 2005, the sentinel system even expanded to 80 sites across China with hilly areas now representing the most sentinel sites. Furthermore, in 2011, diagnostic protocols changed to multiple rounds of diagnoses with the addition of a miracidium hatch test on 30 g of human stool; and the coverage has extended to 458 sites, since 2014. In 2015, the examination of wildlife feces to detect environmental infection risks and the adoption of the loop-mediated isothermal amplification (LAMP) technology were further added. The latter is an effective and sensitive approach for the rapid extraction and amplification of snail genomic DNA to achieve early detection of *Schistosoma* spp. Contrary to microscopic diagnostics, prepatent snails can be discovered as of day one after exposure to miracidia with this test. Both the examination of wildlife feces and the LAMP assay are currently used to generate risk maps that can be applied to guide further local investigations and increase snail control activities in case infected snails are found [19]. In conclusion, this constantly adjusted sentinel system is still ongoing and represents the endemic status of an entire country due to consistent data collection and analysis. It must be noted that it is sometimes necessary to substitute sites in case a population exhibits fatigue from yearly surveys or when prevalence has decreased dramatically [22].

In 2005, Wu et al. described the results from a provincial surveillance system set up in five provinces of China (Fujian, Guangdong, Guangxi, Shanghai, and Zhejiang) with interrupted transmission from 1985 to 1995 (Figure 2). The frequency of snail surveys in formerly *Oncomelania hupensis*-ridden areas was determined by the absence or presence of snails in the past 3–15 years. In villages where snails had been found in the past 3 years, snail surveys were carried out annually. In villages where snails had been found in the past 3–9 years, snail surveys were carried out once every 3 years. In villages where snails had been found in the previous 10–15 years, snail surveys were carried out once every 5 years. Finally, in villages where no snails had been found in the past 15 years, snail surveys were not carried out regularly (i.e., not planned). Additionally, regular snail surveys were carried out in waterways connected with neighboring *O. hupensis*-infested areas, areas where water plants are bred, docking areas with boats, and areas with domestic animals having access to snail-infested areas. In case snails were found, all humans older than seven years and all bovines in these areas were screened first with IHA or ELISA and subsequently, in case of a positive serological test, examined with the KK technique for the presence of eggs in their stool. PZQ was administered to those with one or two positive results. Furthermore, migrants from endemic areas and suspected schistosomiasis cases (i.e., people with fever of unknown cause, symptoms of hepatosplenomegaly, and mucous or bloody stool) were submitted to either one or both of the abovementioned tests and treated with PZQ if they tested positive, and their medical history was traced. The results in 2002 demonstrated that the great achievement of disease elimination had been sustained, with only three human cases being detected since 1992. Nevertheless, as was concluded from the abovementioned national surveillance system, residual snails still existed in areas in close proximity to endemic areas and therefore, China carried on snail surveillance and control [23].

Third, routine surveys are another category of surveillance system, that began in the PRC in 2005, which includes case reports and case surveys across the country (Figure 2). Additionally, routine surveys on human and mammalian hosts in seven endemic provinces are conducted over the course of two or more years depending on the transmission status (control or elimination). At first, case reports and case surveys comprise the reporting and investigation of a schistosomiasis case within 24 h after diagnosis through NIDRS established by the CDC (Section 3.1.3), including detailed information of the case (e.g., name, age, gender, address, contact information, and occupation). Secondly, for counties from endemic provinces that achieved control and elimination, all villages are currently surveyed over a three-year period and five-year period, respectively. An exception is made for villages where information is shared from the national sentinel surveillance system. The routine surveillance involves sampling of humans (>90% of residents between 6 and 65 years old), sampling of cattle and other alternative mammalian hosts, and snail surveys in the spring. Although routine surveys provide spatial and temporal data on infection patterns, some challenges present in the national and provincial surveillance systems still need to be overcome. At first, variations were noted in sampling and screening protocols (although they were standardized by the Ministry of Health) leading to difficult comparison of data between counties. Secondly, survey efforts of the mammalian host are the responsibility of the local animal husbandry department; however, coordination between them and the public health authorities was highly inconstant. At last, participation fatigue often occurred, although this was resolved by the conduction of health education campaigns emphasizing the consequences of chronic, undetected infections and the importance of routine examinations [15,22].

A final category of surveillance is consolidated surveillance in areas where schistosomiasis has been eliminated, but where a risk of re-emergence is plausible, i.e., risk surveillance (Figure 2). The choice of risk surveillance sites relies on results from previous routine surveillance and the availability of local capacity to carry out surveillance. Risk surveillance mainly focuses on the survey of infected water with sentinel mice (see Section 3.1.5), snail investigations, human case detection, and free-roaming livestock surveillance [15]. An example of this is the risk survey conducted between 2008 and 2010 in four counties of three at-risk provinces (Jiangsu, Shandong, and Anhui) in China. Local residents aged between 6 and 65 years were serologically tested using IHA and subjected to fecal examination by KK for confirmation in case of a positive result. Snail surveillance included the capture of snail in frames placed along study sites connected to water bodies and the active search for snails in environments with mobile populations, ships, or plant matter in the water. At last, 30 farms per sentinel site were selected for survey on their livestock and dogs with the miracidium hatch method. During this survey, no snails or positive animals were found, yet 22 positive human cases were confirmed—all of them fishermen, boatmen, or immigrants (i.e., mobile population) [24]. A similar risk surveillance held between 2008 and 2012 also detected 28 positive cases in the mobile population, while no snails were found and neither local nor imported livestock was found to be infected [25]. Both of these results emphasize the important contribution of mobile population to the transmission of schistosomiasis and the importance of monitoring them.

In brief, the PRC is one of the pioneers in the control and elimination of schistosomiasis with sustained M&S taking place in different parallel systems across the country (Figure 2). Therefore, the epidemiology of schistosomiasis has shifted over time and place, as did interventions and strategies. Surveillance systems should now prioritize more the identification of remaining and/or re-emerging hot spots and the evaluation of underlying risk factors and their relation to transmission patterns. In the meantime, China’s acquired experience with M&S and knowledge on diagnostic tools and techniques has the potential to be transferred to endemic countries with limited resources. 

#### 3.1.7. Monitoring and Surveillance for *Schistosoma mansoni* in Egypt

Another pioneer in the control and elimination of schistosomiasis is Egypt, due to its effective monitoring and treatment system. In 2017, this country started a national schistosomiasis elimination plan supported by the World Health Organization after 5000 years of endemicity. Since the implementation of different control strategies in 1922, schistosomiasis (*S. mansoni*) prevalence had substantially decreased to 0.2% in 2016 in most of the endemic areas. The first step from this 2016 control stage to elimination included the re-mapping of residual distribution of schistosomiasis in all endemic governorates using both the KK technique and a urine circulating cathodic antigen (CCA) test [26]. So far, 35 districts of 5 governorates have been re-mapped, while the survey of other governorates is still ongoing. Specifically, a number of schools in rural areas of the districts of each governorate were selected to provide urine and stool samples. These were collected from 100 children per school and tested for schistosomiasis with the KK technique and CCA test. Based on the outcome of the prevalence, a mass treatment policy with PZQ was implemented (i.e., (i) mass drug administration (MDA) of school children in districts with KK = 0% and CCA > 0%, (ii) MDA of all villages in districts with KK > 0% and <3% and CCA > 0%, and (iii) MDA of all rural areas in districts with KK ≥ 3% and CCA > 0%). Additionally, mass treatment of areas included the application of molluscicides and the treatment of water bodies. Further monitoring and treatment of the other governorates will enable the mapping of residual infected areas and the achievement of nationwide elimination of the parasite [27]. Although this represents an efficient system for achieving elimination as well as monitoring at the post-control stage, it must be emphasized that a rigorous post-intervention surveillance system will have to be established to maintain this elimination status. The surveillance system should specifically focus on high-risk areas/individuals, include continuous monitoring of the intermediate snail host and people, and finally, comprise an appropriate response procedure in case infected individuals/snails are found.

#### 3.1.8. Future Challenges and Recommendations for Schistosomiasis

Although mostly occurring in developing countries, the geographical range of schistosomiasis is also spreading toward the Western world due to an increasing number of immigrants and changing pattern of travelers. Specifically, migrants/travelers who acquired the infection externally might in turn introduce local transmission of schistosomiasis in case a suitable intermediate snail host is present in non-endemic areas (e.g., Western Europe, but also in non-endemic areas in China). At the same time, a large proportion of infected people returning/migrating from *Schistosoma*-endemic areas are asymptomatic, which facilitates local transmission introduction even more [28]. Therefore, surveillance and intervention of migrants should be included and strengthened in any schistosomiasis control program, as they may form a new source of infection in non-endemic areas or areas where transmission had been controlled or even eliminated. For example, the results of a study conducted in the endemic Dongting Lake region of China demonstrated a schistosomiasis prevalence rate twice as high in immigrants as in permanent residents. These infected immigrants may move to areas where transmission control/interruption has been achieved, but where snails are still present. This may result in the re-emergence of transmission in these areas given that migration was not a feature of the national control program [29]. Consequently, surveillance systems are to be adjusted to migrants and travelers, for instance, by examination and treatment after water contact, pretravel education, and universal health coverage of the migrant population. Even when the intermediate snail host species is not present in a non-endemic area, schistosome hybridization has been shown to occur and widen the range of snail host species. Subsequently, atypical snail hosts may potentially increase the transmission risk of *Schistosoma* spp. in the non-endemic area. This was the case in Corsica, where *S. haematobium* had been introduced by people travelling from West Africa and had hybridized with the cattle schistosome (i.e., *Schistosoma bovis*). Due to this hybridization, the *Bulinus truncatus* snail, that is widely present in Corsica, became a suitable intermediate host and further aided in the rapid colonization of urogenital schistosomiasis in this country [30].

Other threats for the appearance of new foci or re-infection risk in previously controlled areas are the constantly changing ecological, environmental, and human behavioral factors due to water development projects. These are leading to a higher and changed complexity of transmission-related problems and therefore, result in additional challenges for the control and M&S of diseases. An example of this is the construction of the Three Gorges Dam, the world’s largest hydropower project, in China. Along with its social and economic benefits came dramatic ecological changes resulting in the fact that *Oncomelenia* snails, the intermediate host of *S. japonicum*, could live and breed within the reservoir area. Additionally, an increase in employment (and consequently immigrants) and the transport of products to this area had the potential to aggravate the spread of schistosomiasis in this region [31]. Another example is the construction of the Diama Dam in Senegal, which instigated the spread of schistosomiasis in 1989 [32]. The latter specifically resulted in the detection of *S. haematobium* and an increase of *S. mansoni* prevalence (from 10% to >80%) in the dam region due to severe adverse effects in the local ecosystems. With this in mind, a strategic plan, including environmental assessment and surveillance of livestock (for the zoonotic *Schistosoma* spp.) and human cases in addition to monitoring the geographical distribution of the intermediate snail host, is highly recommended and essential when introducing dramatical environmental changes, such as large-scale water development projects.

Finally, although the improvement of surveillance is crucial to sustain a control/elimination status, there is currently no systematic evaluation of surveillance systems. This evaluation mainly relies on collecting and summarizing data to determine surveillance sensitivity, cost-effectiveness, functionality, and quality. Every so often, an evaluation of human and animal surveillance methods is performed in areas where schistosomiasis has re-emerged, yet these evaluations are not systematic. Therefore, in the future, a rigorous monitoring network should be established that enables a systematic evaluation of the current schistosomiasis surveillance systems in order to improve their accuracy and speed of detection [33].

### 3.2. Soil-Transmitted Helminths

#### 3.2.1. Introduction

STH infections, with a focus on the four major parasite species *Ascaris lumbricoides* (roundworm), *Trichuris trichiura* (whipworm), *Necator americanus* (hookworm), and *Ancylostoma duodenale* (hookworm), cause extensive socio-economic and public health problems and mainly affect people living in areas with poor water quality, sanitation, and hygiene. In addition, travel to/migration from such areas increases the potential for dispersal of STH infections to more developed nations. Transmission occurs when eggs from infected people are passed in the feces and contaminate the soil, water sources, or vegetables. In the case of roundworms and whipworms, their eggs get ingested and adult worms develop in the intestines. On the other hand, eggs from hookworms in the soil release larvae that can actively penetrate the skin. Subsequently, people get infected by walking barefoot on the contaminated soil. Considering that these helminths impair the nutritional status of their hosts and (pre-)school-aged children predominantly carry the burden of this infection, they often present with growth retardation, impaired cognitive development, and anemia as a result [34].

Ideally, the building of decent sanitation infrastructure worldwide could interrupt the transmission of and eliminate STHs. In reality, resources required to sustain this are rarely available. Therefore, the current goal of the WHO for 2020 is to eliminate morbidity rather than the parasites themselves by the administration of large-scale periodic preventive chemotherapy and health education to all populations at risk of developing illness (i.e., (pre-)school-aged children and pregnant women) with a national coverage goal of 75% in endemic countries. Once a country has reached the stage of childhood morbidity control/elimination, the objective is to maintain and refine monitoring/surveillance and to sustain deworming programs which will be discussed below [35]. 

#### 3.2.2. Monitoring and Surveillance of Soil-Transmitted Helminths

After 5–6 years of MDA with good coverage (i.e., >75% of at-risk population), a reduced prevalence and morbidity of STHs should be noticed in the target population. The questions to the public health authorities should then include whether this reduction will be permanent, whether and how the frequency of deworming interventions can be reduced, and whether sentinel site monitoring should be continued. Considering the objective of this review, we will only focus on M&S when morbidity control/elimination has been reached. The latter entails three options: (i) morbidity is under control, yet the risk of re-emergence is high (i.e., prevalence between 10% and 20%), (ii) morbidity is under control and the risk of re-emergence is low (i.e., prevalence between 2% and 10%), and (iii) elimination of morbidity (i.e., prevalence < 2%). According to the WHO, the frequency of anthelminthic treatment can be reduced to once a year for the next four years in the first case. In the second case, the frequency of anthelminthic treatment can be reduced to one round of anthelminthic treatment every two years and in the final case, preventive chemotherapy is not needed anymore. Nevertheless, in all events, continuous annual monitoring is crucial in order to inform control program managers about any possible morbidity recrudescence. If monitoring indicates that prevalence remains low (i.e., between 10% and 20%, between 2% and 10%, and < 2%, respectively, for the first, second, and third morbidity control/elimination options) for 4 years despite the reduced frequency of MDA, a further reduction is in order. In case prevalence tends to re-emerge, the original treatment frequency is warranted [36].

The abovementioned monitoring requires periodic sample collection in order to obtain epidemiological data and to assess morbidity status. To do so, several approaches have been proposed, each with its own advantages and pitfalls (Table 2).

The first and the most commonly implemented approach is the simple collection of samples at sentinel sites. These predetermined sites are located in every homogenous zone of a district or country and are assumed to provide information regarding the prevalence and intensity of infection in the entire area. For example, ever since successful STH control was achieved in 2004 in The Republic of Korea through continued parasitic control projects, national surveys on the infection status of STHs have been carried out in several counties at different time intervals. Every 5–7 years since 1971, a nationwide survey is held to monitor the infection rate and its relation to various characteristics (e.g., age, gender, region, and urban versus rural areas). Each time, 300 districts from a total of 23,536 districts among the country are randomly selected to be sampled. Specifically, people of any age in the selected districts are subjected to fecal examination and subsequently, the results are compared with those from previous surveys [37]. The data showed a dramatic decrease of egg-positive rate from 84.3% in the 1st survey in 1971 to 2.6% in the 8th survey in 2012. Nevertheless, infections with *T. trichiura* are gradually increasing and thus it is deemed necessary to carefully and continuously monitor these infections. In such cases, nationwide surveys do not provide accurate data regarding parasite prevalence compared to surveys on a regional basis. Therefore, studies of inhabitants in areas where STHs are suspected to be present as endemic foci are in place. One article described a similar regional survey in a previously high- and previously low-endemic county in The Republic of Korea to monitor the current status of prevalence of STHs. From May to July 2017, 2033 fecal samples were collected at 27 elementary, 10 junior high, and 8 high schools from two counties and examined for eggs with the KK technique. Results showed very low rates (0% and 0.07% for the previously low- and high-endemic counties, respectively) [38].

A second approach that has been implemented in Sri Lanka and Kenya is the integrated school-based M&S for both lymphatic filariasis (LF) and STHs. Considering that control interventions for different NTDs often overlap (e.g., MDA of albendazole is efficient against both STHs and LF), the opportunity arises to integrate their M&S in order to reduce their costs, including costs for administration, personnel, and transport. Therefore, post-MDA M&S with transmission assessment surveys for LF can integrate the collection of stool samples for STH screening. Although this system is likely more cost-effective compared to parallel programs, it may present some problems. At first, the collection and transport schedules of blood (for LF) and stool samples require some adjustments in order to avoid intervening with each other. Furthermore, caretakers used to handle blood samples may refuse to handle stool samples. Finally, it might be required to streamline sampling strategies for both the parasitic diseases in case a different number of samples from a different number of sampling sites is required. Therefore, further research is needed to integrate the two M&S systems without compromising their integrities [39,40].

Another method for sample collection suggested by Jex and colleagues (2011) is lot quality assurance sampling (LQAS), a minimalistic approach of sampling originally designed for manufacturing inspection. The basis entails the collection of a small number of batches, their control and statistical assessment, and finally, the addition of more batches until statistical significance has been reached. In the context of disease monitoring, this is especially useful in many low-income countries where sampling size is often determined by financial and logistical constraints rather than on a scientific basis. The best sampling plan would associate the lowest sample size at the highest statistical significance, which is especially valuable when a large number of communities needs to be tested under scarce resource availability. The latter can be determined by using tables in which sample size is shown for different type I errors. Although LQAS represents a promising, effective, and cost-efficient approach for monitoring STHs and a wide range of other parasites and pathogens, it has not been implemented so far in any STH control program [34].

Pooling of samples is the final suggested system to estimate the prevalence of STHs, while minimizing the number of diagnostic tests performed. This pooling process involves the aggregation of equal proportions of a number of samples to make up one pool. Subsequently, one single test allows to measure the properties of all constituent samples and therefore, fewer tests are necessary when using a larger pool size. Furthermore, in case one pool tests negative, all the constituent samples are negative. On the other hand, in case one pool tests positive, each included sample is tested individually in order to identify the infected individuals. This system definitely represents an appealing approach that significantly gains in time (e.g., with regard to microscopic examination techniques) and budget (e.g., with regard to molecular tools) in low prevalence regions [41]. Nevertheless, according to a study from Leta and colleagues (2018) in Ethiopia, a pooled strategy reduces the detection sensitivity due to a dilution effect and only an 11% reduction in total operation costs was obtained compared to individual examination. In sum, for future application of this pooled approach, further studies are required to determine its advantages and disadvantages at different scenarios of prevalence and diagnostic tests used [42].

It can be concluded that a number of methods for the collection of epidemiological data regarding STHs are possible; however, data collected using different approaches are often hard to assess with standardized methods. Differences in sampling protocols, timing of collection, and sample sizes limit the utility of the data for cross comparison among regions. Moreover, cross comparisons often do not comply with the requirements needed to obtain statistical significance due to these differences. Therefore, validating and standardizing M&S protocols in addition to sharing of epidemiological databases need to be in place in (i.e., on a national level) and between (i.e., on a global level) STH-endemic countries.

### 3.3. Echinococcus *spp*.

#### 3.3.1. Introduction

Echinococcosis is a widespread helminthic disease affecting humans and is transmitted by domestic and wild animals. The two species of the *Echinococcus* genus of major public health importance are *Echinococcus granulosus* and *Echinococcus multilocularis*, both of which cause serious morbidity and mortality in humans if left untreated. Cestode eggs excreted by the definitive hosts (primarily dogs for *E. granulosus* and foxes for *E. multilocularis*) are ingested by the intermediate hosts (herbivores for *E. granulosus* and small rodents for *E. multilocularis*) in which the hydatid/alveolar cysts develop. Subsequently, transmission is completed when the intermediate host (infected organ) is ingested by the definitive host resulting in a tapeworm infection. Humans can also act as an accidental intermediate host through contact with dogs/foxes or contamination of food and water with infective eggs from feces [43].

Despite some progress in the control of echinococcosis, effective control has not been achieved or even attempted in most endemic countries. Furthermore, positive achievements of control programs at the local level have not markedly changed the global health importance of the disease. The development of effective control strategies and the management of livestock and ecology of dog and wildlife definitive hosts remain a challenge. Therefore, many articles are concentrating more on control interventions or surveillance in countries previously thought to be free of echinococcosis. The latter resulted in a rather low outcome of the search strategy for articles focusing on post-control/elimination M&S of *Echinococcus* spp. Additionally, two articles were included focusing on surveillance in Sweden (where echinococcosis is emerging) and Japan for the reason that these systems would also be effective at the control/elimination stage (Table 3).

#### 3.3.2. Monitoring and Surveillance of *Echinococcus* spp.

The first, often implemented, M&S method for *E. granulosus* is the routine post-mortem meat inspection (MI) at the abattoirs. This method is applied in many countries, such as Iceland, where *E. granulosus* has been gradually controlled since 1894. It represents a useful tool to assess the national and regional animal health status and to provide farmers with feedback on the infection status of their animals as can be illustrated by the following article. The article described the abattoir surveillance system for 352,325 Tasmanian sheep throughout a 5-year recording period (2007–2013) and stated a prevalence of 0.01%, which highlighted the purposes of a hydatid cyst control program operating in this country since 1965. Subsequently, in case of positive results, mandatory sampling in addition to treatment with cestocidal drugs (of shepherd dogs) at the farm of origin was applied. Although reliability studies state that trained inspectors can correctly identify most lesions during MI, there is potential for over- or under-estimation of prevalence. This points out the need for further studies regarding meat inspector diagnostic sensitivity and differences between abattoirs. Furthermore, this passive abattoir surveillance system is rather time-consuming and highly dependent on the correct ear tag identification of individual sheep (or cattle/pigs) for accurate farm tracebacks. Therefore, this method should never be implemented as a stand-alone system, but should always be combined with other veterinary surveillance strategies and continued public awareness campaigns. Nevertheless, despite its shortcomings, the findings of this study support the purpose of ongoing routine MI and on-farm sampling and treatment activities [44].

Secondly, three articles described M&S with regard to *E. multilocularis*. Until 1980, only four European countries were known to be endemic for this fox tapeworm; however, since then *E. multilocularis* has been increasingly spreading, with its presence confirmed in foxes currently in at least 24 countries of the European Union. Among these countries is Sweden, originally free from echinococcosis (i.e., a prevalence of < 1% in the final host), where a surveillance program was initiated in 2000. The objective was to sample 300 red foxes annually from each municipality in order to estimate the prevalence and geographical extent of the infection across the country. Hunters submitted foxes (against a small remuneration), which were subsequently examined by the sedimentation and counting technique (SCT) and real-time PCR. Since the two positive findings in 2011, 140 fecal samples from hunting dogs in the four municipalities around the parish, where the positive foxes were shot, were collected and examined with a copro-antigen ELISA. Additionally, the sampling of foxes was intensified to 3000 annually and diagnosis with SCT was replaced with the more cost-effective, yet equally sensitive, segmental SCT. Rodents and fox feces were also collected for autopsy and analyses by copro-PCR, respectively [45,46]. This annual surveillance system is still ongoing and so far, four infected counties across Sweden have been revealed as having a first infected rodent. These results demonstrated that repeated monitoring of *E. multilocularis* in Sweden is definitely necessary and must even be intensified to clarify whether prevalence is increasing. Another result to the positive findings in Sweden, approximately 60 km from the Norwegian border, was the intensification of the red fox surveillance in Norway. Specifically, 500–600 fecal samples from red foxes were annually collected by hunters across the country and analyzed with copro-PCR. So far, each of these have tested negative, which supports the value of the annual surveillance system [47,48]. Finally, in Japan, the *E. multilocularis*-endemic area is restricted to the northern island of Hokkaido, where control is on its way since the enforcement of the echinococcosis control program. The risk for dog-to-human transmission by eggs excreted by stray and free-roaming dogs remains a major concern. Between 2013 and 2017, 156 shelter dogs were examined using fecal egg examinations and copro-PCR. While the prevalence of the tapeworm in companion dogs was only 0.4% in this region, a 1.9% prevalence was discovered in the shelter dogs as these are more likely to get infected by catching an intermediate rodent host. This demonstrates the purpose of continued preventive deworming of not only companion dogs, but also of stray and free-roaming dogs in order to prevent potential human infection and maintain the status of control [49].

### 3.4. Taenia *spp*.

#### 3.4.1. Introduction

*T. solium* and *T. saginata,* the pork and beef tapeworm, respectively, are the most important causes of human taeniosis (TS). A cysticercus is the metacestode larval stage of these *Taenia* spp. that develops in the bovine or porcine intermediate host after ingestion of eggs (i.e., cysticercosis (CC)). These eggs are shed by the final human host, when the consumption of raw/undercooked infected pork or beef meat results in the presence of a tapeworm in the small intestine (i.e., TS). Although not a serious public threat, the financial burden of *T. saginata* due to carcass condemnation, freezing, and devaluation should not be underestimated. In the case of *T. solium*, humans may also act as an intermediate host when ingesting infective eggs. As such, cysticerci have the potential to develop in the brain (central nervous system), collectively named neurocysticercosis (NCC), with epilepsy, migraine, and other neurological manifestations as a consequence. Furthermore, not only NCC, but also porcine cysticercosis (PCC), has an enormous economic impact due to many related costs, such as hospitalization and loss of condemned carcasses [50,51]. Therefore, the increase in public awareness of these consequences has led to the implementation of prevention and control measures (such as pig confinement, vaccination, and treatment; human treatment; and health education) against *T. solium* in more and more countries. Additionally, meat inspection, which is achieved through carcass visual inspection and/or incisions, is a fundamental prevention measure for both the *Taenia* spp. and is currently included in many legislations of high- and low-income countries [52]. In sum, many countries worldwide are still considering/aiming for control/elimination of *Taenia* spp., which, once again, resulted in a low outcome of the search strategy for articles focusing on post-control/elimination M&S.

#### 3.4.2. Monitoring and Surveillance of *Taenia solium*

Only three articles described the design of a surveillance system, all in countries where *T. solium* was formerly controlled/eliminated. Nonetheless, at least 15 articles (2 articles included in this systematic review and 13 articles not included) concluded the urgent need for a common strategy on data collection, monitoring, and reporting of *T. solium* cases, in addition to an improvement of the current human and animal surveillance systems, for example, by implementing obligatory notification of cases (Table 4).

The first article by Fonseca and colleagues (2018) described the establishment of an integrated surveillance program applicable to industrialized countries. Due to increased travels and migration, *T. solium* TS and CC are emerging in Europe, where these diseases were formerly under control/eliminated. Additionally, data on prevalence are scarce and non-updated, both of which request for combined approaches and one health solutions. Therefore, Portugal was the first country to design a surveillance system in 2017, named the Observatory of Taeniasis and Cysticercosis. This system comprises several core activities to obtain essential information on the burden and epidemiology of CC in Portugal, namely, (i) the identification of possible geographic hotspots by systematically obtaining and analyzing data from hospitals, slaughterhouses, and human social sectors (the latter with regard to residents and migrants), (ii) the implementation of questionnaire-based surveillance targeting healthcare units and porcine distribution units to detect and monitor new human and porcine CC cases in addition to epidemiologic field surveys exploring human, animal, and environmental factors in order to identify possible human tapeworm carriers, (iii) hospital-based treatment for identified human tapeworm carriers, (iv) monitoring and supervision of the reporting process, and (v) and yearly data dissemination. This system, mainly focusing on the detection and treatment of tapeworm carriers, has just recently started and needs to be run for a minimum of five years to allow complete outcome-based evaluation; however, it may prove a valuable addition in future health programs [53].

In 2016, Priest and colleagues described the opportunities of multiplex bead assays (MBA) to monitor the impact of different NTD interventions and their post-elimination surveillance. Nowadays, multiple indicator surveys are being conducted worldwide to obtain information on a large number of diseases often using a single blood sample; however, these surveys have not been extended to include NTDs. Therefore, the Cambodian Ministry of Health conducted a national integrated biomarker survey in 2012 to gather not only information regarding immunity for poliomyelitis, measles, rubella, and tetanus, but also for several NTDs, among them *T. solium*. Samples were collected and tested by MBA for immunoglobulin G antibodies in order to generate national prevalence estimates for parasitic and viral diseases of interest. Despite its tremendous potential, multiplexing technology for integrated surveys also has its shortcomings. First, defining seropositivity cutoff values for some antigens can be challenging when banks of positively and negatively confirmed cases are not readily available. Second, cross reactivity due to previous exposure to other helminth parasites may result in false-positives, although this can be assuaged by including multiple parasite antigens in the MBA. At last, the surveys are often powered to compare regional prevalence estimates, considering the different expected levels of the diseases included. In this matter, estimates at the province or district level are missing, yet nationwide hotspots can be identified and used to target further focal screening surveys. Despite these limitations, the integration of MBAs for parasitic diseases, such as *T. solium*, into multiple indicator surveys definitely provides a useful tool for monitoring and evaluating control interventions and post-elimination surveillance for many diseases [54].

Finally, the third study implemented a ring screening and treatment system in northern Peru to reduce *T. solium* transmission in resource-poor communities. Specifically, all pigs of the intervention village (1058 residents) were surveyed for PCC by tongue inspection every four months. Whenever a heavily infected pig had been found, residents living within a 100 meter radius were tested using a copro-antigen ELISA and treated with a single oral dose of niclosamide in case of a positive result. Although the ring screening and treatment system had been used as a low-cost control measure in this study, it was stated that the ring strategy could also potentially be applied as a surveillance system at the post-control/elimination stage [55].

#### 3.4.3. Monitoring and Surveillance of *Taenia saginata*

As is the case for *T. solium*, the control of *T. saginata* is also still far away from being realized due to a variety of factors and therefore, most articles focus on control and prevention measures rather than M&S (Table 4). Since the 64/433/EEC directive of 1964 (now EU 854/2004, and EU 625/2017), MI of all bovines over 6 weeks of age (whereby the predilections sites for CC are visually examined and/or incised to detect cysticerci) is the cornerstone for the control and surveillance of *T. saginata* in Europe. In case of general infection, the carcass is declared unfit for human consumption. In case of localized infection, the carcass must be stored at –10 °C for >14 days before release. Although widely implemented with moderate to high specificity, MI results in an underestimation of the prevalence of CC by a factor of 3–10 due to its low sensitivity [56]. Moreover, a study from Jansen and colleagues (2017) recorded an underestimation by a factor of 100 and a sensitivity of <0.76% for MI in Belgium [57]. At first, motivation and skills of the inspector play a huge role, as does the stage of degeneration of the cysticerci. Furthermore, in lightly infected animals, which is the biggest proportion of infected animals in Belgium, only a fraction of the cysts were found in the predilection sites [58].

Given the fact that current post-mortem MI is limited by its poor sensitivity, alternative methods have been studied thoroughly. In 2016, Marshall et al. suggested the implementation of risk classification of farms/cattle and slaughterhouses based on data from history cases, serological tests, and other known risk factors (e.g., gender, age, presence of contaminated pastures and water sources, and movement history) in order to improve conventional MI. For example, it was stated that young male cattle are less likely to be infected, whereas cattle derived from farms where an infected animal has resided before are at higher risk of infection [59]. Therefore, visual inspection on low-risk cattle and rigorous MI on high-risk cattle makes this surveillance system more targeted and effective compared to conventional MI [60,61].

### 3.5. Integrated Monitoring and Surveillance of Neglected Tropical Diseases

Although many articles mention the need for integrated M&S of several pathogens, only three records explicitly described a design of such surveillance for the helminthic zoonoses discussed above (Table 5).

One author suggested an integrated M&S system of both *T. saginata* and *E. granulosus* in hypo-endemic countries based on the identification of risk areas by a spatial analysis. A study on this was carried out in the Veneto region (northeastern Italy), where the prevalence of bovine CC and cystic echinococcosis (CE) is generally low. The data (age, sex, location, etc.) on local farm cattle slaughtered between 2006 and 2010 were integrated with passive abattoir surveillance data on the bovine CC/CE status of each animal using geographical information systems. Subsequently, the aggregation of this data was investigated using a scan statistic with a Bernoulli probability model in order to locate the most likely bovine CC and CE clusters. Its reliability with regard to CE was confirmed by an epidemiological survey collecting and analyzing dog fecal samples from all farm dogs located in a cluster area. Additionally, based on the identification of the clusters, detailed epidemiological surveys on positive farms in these areas could contribute to the clarification of risk factors maintaining this disease and the identification of the most probable introduction route of *T. saginata* or *E. granulosus* into the farms. Concerning surveillance response, further actions can be taken based on the outcome to break the parasite’s life cycle (e.g., deworming of dogs, examination and/or anthelminthic treatment of farmers, intensified on-farm control, and a more rigorous MI of farm animals derived from such clusters in order to substantially increase the sensitivity of detection). It can be concluded from this study that a retrospective approach of the spatial analysis for integrated surveillance is very effective, especially for these two parasitic diseases considering their mandatory abattoir surveillance [62].

The second article described the implementation of cross-sectional surveys in Taboo (south-central Côte d’Ivoire), where morbidity control of STHs and schistosomiases was achieved due to preventive chemotherapy. Additionally, an anamnestic questionnaire was administered with the ultimate goal to estimate the individuals’ helminth infection status and subsequently apply a more cost-effective targeted treatment. Seven percent of all the households in the study area were selected for a random sampling procedure (i.e., stool and urine for microscopical examination with KK and CCA tests) and completing a questionnaire on risk factors and signs and symptoms pertaining to different NTDs, among them STHs and schistosomiasis. Subsequently, multivariable logistic regression was used to reveal associations between the infection status of different helminths and the diagnostic variables (i.e., risk factors, signs, and symptoms). Disappointingly, results showed that risk factors, signs and symptoms should not be considered as strong diagnostic variables as they did not perform well in predicting schistosomiasis and STH infections. Therefore, the question arises to how one might identify groups that need treatment when targeting multiple NTDs. Despite the shortcomings of anamnestic questionnaires for both STH and schistosomiasis, efforts have already been made to integrate monitoring for these two parasitic diseases and this opens the way for the development of more comprehensive approaches for M&S of several NTDs [63].

Finally, the third article studied the potential of a polio surveillance system for the screening of STHs and schistosomiasis at the same time. Since the WHO set out to eradicate polio worldwide in 1988, the global polio laboratory network (GPLN) was established with 16 GPLN labs across Africa receiving an average of 22,017 fecal samples per year. Despite its major contribution to the eradication of polio, samples collected here are only screened for polio, while many diseases tend to be co-endemic in the poorest communities. Therefore, the Ghanaian National Polio Laboratory explored whether it is possible to identify polio as well as STHs and schistosomiasis from the collected fecal materials. The samples were linked to the age and region from the participant and tested for the different helminth types using a multiplex PCR assay. The findings of this study showed that it would indeed be sensible to expand the existing surveillance platform of polio to other diseases and include new diagnostic techniques, such as multiplex PCR. Nevertheless, the samples in this study were not representative for the different regions of Ghana as they originated from individuals presenting specific symptoms of polio. With a change in sample submission policy, the GPLN surveillance platform would be suitable for pilot screening of other NTDs and cost-effective compared to a de novo set up of standard surveillance platforms for each parasite separately [64].

### 3.6. Future Challenges and Recommendations

#### 3.6.1. The Case for Strengthening Diagnostic Capacity

As the prevalence of many NTDs decreases, accurate and sensitive diagnosis is vital for M&S, especially in low-endemic regions to assess the impact of control programs, to identify risk areas, and to detect re-introduction or new transmission areas [11,65]. Nevertheless, a fast and standardized diagnostic method suitable for large-scale sampling is often lacking. For instance, the SCT for the diagnosis of echinococcosis was long seen as the golden standard for detection in the final host, yet it represented a labor-intensive and time-consuming method [66]. Furthermore, many diagnostic techniques rely on the determination of eggs in urine for urogenital schistosomiasis (*S. haematobium*) [67] and the microscopic detection and counting of eggs in stool samples, such as the KK technique for intestinal schistosomiasis (*S. mansoni* and *S. japonicum)* and STHs [68,69]. These techniques are highly specific (except for *Taenia* spp.), easy to perform, and relatively inexpensive. Nevertheless, issues with sensitivity are well-known and may result in an underestimation of the prevalence, particularly in countries that have reached the control/elimination stage. As a result, the obtaining of an additional fecal sample (i.e., duplicate KK technique) has been suggested, as this substantially improves the diagnostic sensitivity (23–100%) despite the increase in cost (31%) [68]. On the other hand, the FLOTAC technique (i.e., another fecal egg count technique) offers a higher sensitivity than a triplicate KK examination as it uses a much larger amount of stool, thereby increasing the chance of detection [70]. Nonetheless, all the above-mentioned techniques have the major limitation that they require trained personnel to identify eggs and tapeworms at species level and to distinguish eggs from other microscopic features.

During the past 15–20 years, substantial progress has been made with immunological methods detecting circulating antigens and antibodies of several NTDs. Two examples of such immunological diagnostic tools are the up-converting phosphor lateral flow assay detecting the *Schistosoma* CCA in body fluids and the point-of-care CCA test detecting a different *Schistosoma* antigen in urine [71,72,73]. Both the tools display advantages over microscopic detection and have proven to be rapid, cost-efficient monitoring tools to indicate worm burden. Other promising and often implemented immunological diagnostic tools are the copro-antigen ELISA for the detection for *Echinococcus* spp., and (cocktail-)ELISA, magnetic affinity ELISA, IHA, and dot immunogold filtration assay for the diagnosis of schistosomiasis. Finally, several rapid diagnostic tests targeting the antigens of or antibodies against foodborne helminthic zoonoses have been developed (e.g., the rES33 magnetic immune-chromatographic test and the up-converting phosphor reporter rT24H lateral flow assay for *T. solium*, the HP10 lateral flow assay for *Taenia* spp. and *E. granulosus*, and HCF-rEM18-immunochromatography for *Echinococcus* spp.). These qualitative or semi-quantitative diagnostic medical devices have been designed to give fast results without the involvement of any automated procedures. Despite their potential use as tools in the M&S of control programs, especially when integrated with conventional tests, many of these rapid tests have not yet undergone any field performance evaluation [74]. With some validation and optimization, these tools definitely present a huge step forward in the M&S for zoonotic parasites in multiple host species [75,76,77,78,79].

Although there is increased development of immunological diagnostic tests, it must be noted that their sensitivity does not yet reach that of molecular diagnostics [80,81]. As a solution, the replacement by highly sensitive and specific molecular diagnostic tools has been suggested to tackle this problem of sensitivity and to perform insightful epidemiological studies [82]. Nevertheless, their use is currently still hampered due to the need for trained personnel, expensive laboratory equipment, and cold-chain storage, often not available in endemic countries with low-resource settings. For example, PCR is the gold standard for several pathogens in terms of specificity and sensitivity, but is relatively expensive compared to the KK method [75,80,83,84,85,86,87,88]. Another recently developed, promising, sensitive, and specific molecular diagnostic tool is the 28S lateral flow recombinase polymerase assay for schistosomiasis, which requires less equipment, specialized personnel, and technical support, but still needs some further optimization and validation before it can be implemented in the field [88,89]. Finally, current studies evaluating LAMP assays for the early detection of *Schistosoma* and *Echinococcus* DNA in stool and serum samples reveal easy to perform, specific, and sensitive diagnostic tests [74,90,91,92]. In summary, molecular diagnostics are currently unlikely to be implemented in large-scale control programs considering their cost; however, with some field-friendly adaptations and standardization of these tests, they are definitely applicable as a future field diagnostic and surveillance tool for low transmission areas [66,78,81,93]. 

In conclusion, the constantly growing control efforts against NTDs urgently require research and further investment into improved and standardized diagnostic tools, especially at the stage of control/elimination. When applying some adjustments with regard to standardization and validation, the recently developed tests discussed above definitely offer field-deployable potential for the future [74]. Furthermore, their potentially higher costs may be outweighed by their long-term benefits [69,94,95].

#### 3.6.2. The Emergence of Drug Resistance

Although no evidence of drug resistance has yet been confirmed, its risk is emerging considering the large-scale implementation of broad-spectrum anthelminthic drugs for STHs and schistosomiasis. So far, many studies regarding the efficacy of drugs have been performed; however, all of which are highly heterogenous in their reporting of results, design set-up, and implementation, making it difficult to draw any conclusions [96]. Therefore, the establishment of a standard operating procedure for the periodic monitoring of anthelminthic efficacy is in place. For example, the application of statistical modeling on a shared database containing individual patient data on drug efficacy against not only STHs and schistosomiasis, but also other NTDs, shows great potential for public health end-users to identify atypical responses potentially indicative of emerging drug resistance [97,98]. 

#### 3.6.3. The Introduction of Spatial Technology

As mentioned above, (re-)emergence of many parasitic diseases is frequently associated with human factors, such as travel/migration, trade, and global population expansion. Furthermore, due to the often complex ecology involving multiple hosts and transmission routes, many diseases are affected by the advent of climate change and therefore, tend to expand their geographical distribution in the future. Finally, to achieve nationwide disease elimination, rapid detection of remaining sources of infection in low-endemic areas is essential. Although mass surveys would provide valuable data in this context, these are often not evident in financial and practical terms. Therefore, changes in the current applied M&S strategies are definitely required. This generates a chance for the introduction of spatial technology in M&S systems in order to improve survey quality and meet the requirements of current monitoring work [99]. 

Spatial technology includes any of the formal techniques which study the description and examination of diseases and their geographical variations by using data collected during previous surveys, i.e., geographical information systems (GIS), remote sensing (RS), global positioning system (GPS), Google Earth (GE), and spatiotemporal statistics and models [100]. The combination of land surface information (soil type, altitude, vegetation index, etc.) derived from RS and GE, weather conditions, GPS coordinates, and direct data from M&S sites (e.g., infection rate) can be used to spatially analyze complex geospatial data and to construct maps showing not only high-risk areas, but also the scope of disease/intermediate host distribution. These maps can be considered as an innovative tool to further guide risk-based surveillance strategies [18,101,102,103,104]. Finally, the application of the aforementioned tools combined with statistical or mathematical modeling provides important advances to explore spatiotemporal transmission dynamics, trends in habitat range, and risk/environmental factors of diseases in the past, present, and future [105,106,107,108]. This can consequently guide surveillance systems and point out areas that still acquire continuous monitoring. Nevertheless, these rapid and cost-efficient tools can be used at any stage of a control program (e.g., to establish baseline estimations, predict the spatial parasite distributions, and select treatment strategies), especially in regions with limited available epidemiological data.

For the M&S of schistosomiasis at the post-control/elimination stage, 15 articles obtained in this review indeed mentioned the use of spatial technology, out of which the majority focused on using it for monitoring snail density [12,15,18,99,100,101,103,104,105,106,107,108,109,110,111]. At present, snail surveys are extremely time-consuming as they are conducted manually and their quality can be influenced by many factors, such as temperature, light, and vegetation. Spatial technology takes into account the interplay between the snail intermediate host and environmental factors and therefore, helps the targeting of surveys. Nevertheless, it must be noted that transmission does not solely depend on the distribution of snails. Therefore, more studies are required that also put emphasis on the infection status of snails and other important reservoirs (such as bovines, dogs, people, and water bodies) that include certain human behavior as a risk factor and investigate access to water of each intermediate host. 

Similarly, at least five records suggested the application of spatial technology at the post-control/elimination stage of STHs [34,112,113,114,115], while four articles did this for both *E. multilocularis* and *E. granulosus*. Specifically, for echinococcosis, an opportunity for landscape epidemiology was recommended by these articles with regard to surveillance. This landscape epidemiology investigates environmental and anthropogenic factors (e.g., temperature, rain fall, urbanization, deforesting, and agricultural practices) and makes integrated use of spatial technology in order to define the link between these variables and disease risk and to design more accurate predictive transmission models and updated risk maps [110,116]. The latter may subsequently guide decision makers to target serological screening of emerging transmission foci identified by these predictive modeling tools. This presents a cost-effective alternative to mass screening in order to detect early cases and has already been successfully implemented in Finland, Ireland, and the United Kingdom [117,118].

Although the implementation of spatial technology for the M&S of many diseases is increasingly being propagated, this spatiotemporal epidemiology is, however, a sophisticated profession in terms of training, expertise, and experience in both mathematical and computational knowledge and often requires expensive specialized software. Fortunately, this is gradually being countered by the provision of free software for mapping, the use of Internet for dissemination of information, and free access to satellite imagery of GE. At last, even the most updated form of spatial technology still requires surveillance data from the field in order to dispense any trustworthy information and conclusions. These data are often lacking with deficient quality or heterogeneity due to varied sampling and diagnostic protocols [119]. As can be seen, spatial technology definitely offers powerful capabilities that facilitate the M&S of diseases, yet more research is required to tackle its related costs and to further guide on-site data collection.

#### 3.6.4. The lack of data for *Taenia* spp. and *Echinococcus* spp.

It can be concluded that many countries are far from reaching a control, let along elimination, status for both *Taenia* spp. and *Echinococcus* spp. This resulted in limited records describing a functioning, validated M&S system at the post-control/elimination stage of these parasites. Moreover, the few methods described above were derived from scattered infrequent surveys as existing epidemiological data on regular national surveillance is lacking. This is especially the case for *T. solium* spp. as this is a non-notifiable disease in most countries and PCC prevalence is based on the underestimating MI [120]. Also, echinococcosis remains a huge challenge considering the asymptomatic infection in livestock and dogs, the involvement of many types of wildlife species in the transmission of *E. multilocularis*, and the fact that it is not prioritized by local veterinary services and public health institutions. Therefore, systematic national and sub-national data collection systems covering all parts of an endemic country are first recommended to reveal the true prevalence profiles before one can even consider sustained control/elimination [58]. 

### 3.7. Limitations of Systematic Literature Review

This systematic literature review has potential limitations. First of all, the process of literature search and study identification was conducted by only one reader, which could have given rise to bias at the study and outcome level. For the same reason, incomplete retrieval of the identified research might have occurred at the review level. Finally, there was no inclusion of grey literature and the review only focused on English literature, which inherently may have resulted in missed information. Despite these limitations, it is believed that their impact on the quality of this review is little given the descriptive presentation of the results and considering that this is the first review, to the best of our knowledge.

## 4. Conclusions and Global Lessons 

Although combined health efforts in the past two decades have led to significant improvements in the battle against *T. solium*, it still poses a major public health threat worldwide. Furthermore, as control and elimination programs are progressing, the sustainability of achieved control and elimination remains their ultimate challenge, which continuously requires rigorous M&S. The latter are essential and integral components of any control program as they provide the ability to take pragmatic action against (re-)emergence, to deliver reliable information on prevalence, incidence, spatiotemporal distribution, and burden of diseases, to identify disease transmission hotspots and risk areas, to evaluate the applied program, etc. Nevertheless, a concept of M&S at the post-control/elimination stage of *T. solium* has not been (fully) developed and validated as can be concluded from this review. Subsequently, when looking into *T. saginata* due to its similarities with *T. solium*, and *Echinococcus* spp., the same conclusion could be drawn. Moreover, reliable estimates on global distribution and transmission of any of these parasitic species are still absent, which needs to be tackled urgently before implementing post-control/elimination M&S strategies. While there still remains much to be done in terms of control of *Taenia* spp. and *Echinococcus* spp., substantial progress has been achieved in China, particularly with regard to STHs and schistosomiasis. How exactly these strategies may be translated to other countries must be explored.

When developing an appropriate M&S framework, it is also important to determine how many animals/people, at how many sites, at what frequency, and at which site need to be collected in order to gain a realistic idea on the disease status and to rapidly detect new cases. Sample size calculations depend on a number of determinants (e.g., variance, prevalence, and consequences of the disease) and may increase so dramatically that they quickly become unrealistic in practical and financial terms. Further research into the development of alternative statistical frameworks for sample size calculations is therefore needed to solve this problem. Additionally, instead of random selection of inspection sites, local knowledge and understanding of site-specific heterogeneities should be embedded in a sampling framework, which provides a unique opportunity for spatial technology. Taken together, sampling should be a semi-structured activity, taking transmission status and the everchanging environment also into account [121,122].

Furthermore, the design of M&S systems for various NTDs vary considerably due to their distinct life cycles and epidemiological features. Still, control and elimination strategies, especially those for STHs and schistosomiasis (e.g., large-scale MDA), have striking similarities which provide opportunities for the possible integration of M&S. Ideally, such integration would include the harmonization of sampling strategies, fieldwork protocols, and diagnostic approaches, which most likely would result in increased population compliance, simplification of survey procedures, and most importantly, reduced costs. Specifically, these diseases tend to be co-endemic in the poorest communities, which would benefit from these financial advantages [123]. Nevertheless, a challenge in the integration of M&S is the establishment of a sampling strategy for diseases that do not perfectly overlap within districts or countries. Consequently, the issue arises in calculating a sample size large enough to obtain a required significance level. Other challenges that need to be addressed are the fact that M&S indicators are often measured in different populations (e.g., school-aged children for STHs vs. communities for schistosomiasis) and the little overlap in diagnostic procedures or sample matrix. In sum, although full integration of all elements of M&S for several NTDs is not feasible, opportunities should be considered for at least some integrated strategies [124].

Next, all the four helminthic zoonoses discussed above are among the most serious helminth zoonoses threatening public health worldwide (except for *T. saginata*); however, diagnostic tools to detect them are still imperfect. Accurate diagnostics are crucial both upstream (e.g., survey and design) and downstream (e.g., targeting, monitoring, evaluation, and surveillance) of control programs. This is especially the case in low-prevalence and low-endemic areas that are approaching elimination, thanks to the continuous implementation of control measures. Furthermore, expanding treatment programs will increase the pressure on drugs for resistance development, which requires monitoring during any operational program, especially those of STH’s. In order to do so, new and sensitive diagnostic tools are once again an urgent need [125].

Finally, considering that not only the NTDs discussed here, but also many other NTDs, have animal origins, a more effective collaboration between medical and veterinary services is required. Currently, many M&S systems consist of separate animal- and human-orientated components, which bring along the challenge to build bridges between human and veterinary medicine in the routes to control/elimination sustainability [126]. Furthermore, both M&S require strong leadership, international cooperation, and the allocation of adequate resources, infrastructure, and qualified staff for the collection, analysis, and reporting of data. Unfortunately, in developing countries, where most of these diseases circulate, these are often poor to non-existent due to budget shortages. Last, but not the least, there is an urgent need to harmonize/standardize M&S activities in order to reliably determine the epidemiological situation worldwide and compare results from different countries. A major step forward in this would be the establishment of a global, constantly updated, NTD database providing open access to every georeferenced survey data.

## Figures and Tables

**Table 1:** Summary of records describing monitoring and surveillance systems for *Schistosoma* spp. at the control/elimination stage.

*Schistosoma* Species	Monitoring & Surveillance (M&S) System	Time	Country	Reference
*S. mansoni*	Malacological survey to determine dispersal: Capture and identification of snails in a radius of 2 m at 168 selected sites, linked to data collection and analysis of water/sediment samples; landscape and climate data.	2016–2017	China	[13]
*S. japonicum*	Surveillance for human schistosomiasis cases: Continuous surveillance of advanced human cases in addition to immunological testing of villagers at risk and suspected patients.	1995–2002	China	[14]
All *Schistosoma* spp.	M&S for human schistosomiasis cases: Reporting of both acute and chronic cases of schistosomiasis diagnosed in hospitals to the National Infectious Diseases Reporting System by all levels of medical institutions.	1989–…^1^	China	[15]
All *Schistosoma* spp.	M&S for human schistosomiasis cases: Self-selected data entry of schistosomiasis cases in the TropNetEurop ^2^ database.	1999–…^1^	Europe	[16]
*S. japonicum*	M&S of livestock: selective treatment or isolation of the infected animal reservoir (i.e., cattle, water buffalo, goats, sheep, pigs, and dogs) to prevent further contamination of the environment.	Not yet implemented	NA	[17]
*S. japonicum*	M&S of *Schistosoma*-infected water using sentinel mice in cages on the water surface.	2008–…^1^	China	[18,19]
*S. japonicum*	M&S of *Schistosoma*-infected water: Detection of contaminated water with environmental qPCR.	2016	Madagascar	[20]
*S. japonicum*	National sentinel surveillance system: Surveillance of humans, cattle, and snails in 20–458 sentinel sites across the country. Treatment of positive humans and cattle, regular re-examination of advanced cases, and focal mollusciciding. Since 2015, additional water bodies and environmental surveillance (i.e., examination of wildlife feces) have been added.	1989–…^1^	China	[15,19,21,22]
*S. japonicum*	Provincial surveillance system (5 provinces): Surveillance of snails (frequency determined on absence/presence in the past 3–15 years). If snails were found, surveillance of humans and bovines, and treatment with praziquantel (PZQ) when positive.	1985–1995	China	[23]
*S. japonicum*	Routine surveys across the country: Case reports and surveys of human patients in addition to regular M&S of endemic villages (humans, snails, bovines, and other mammalian intermediate hosts) that achieved control and elimination.	2005–…^1^	China	[15,22]
*S. japonicum*	Risk surveillance of humans, water, free-roaming livestock, and snails based on results from previous routine surveillance.	2008–…^1^	China	[15,24,25]
*S. mansoni*	National elimination plan based on a monitoring and treatment system: Re-mapping residual schistosomiasis distribution in every governorate of the country with an additional mass treatment (with PZQ) policy based on the prevalence outcome. Additionally, application of molluscicides and treatment of water bodies regardless of the outcome.	2017–…^1^	Egypt	[26,27]

^1^ Still ongoing, ^2^ The European Network on Imported Infectious Diseases Surveillance.

**Table 2:** Summary of records describing monitoring and surveillance systems/sampling methods for soil-transmitted helminths (STHs) at the control/elimination stage.

Method of Monitoring & Surveillance/Sampling	Time	Country	Reference
National surveys (in several counties at different time intervals): The implementation of a nationwide survey every 5–7 years in people of any age to monitor the infection rate and its relation to various characteristics (e.g., age, gender, region, and urban versus rural areas).	1971–…^1^	The Republic of Korea	[37]
Regional surveys: Fecal sample collection of school-aged children in a previously high- and previously low-endemic county in The Republic of Korea to monitor the current status of prevalence of STHs.	2017	The Republic of Korea	[38]
Integrated school-based monitoring and surveillance for both lymphatic filariasis and STHs by fecal examination of school-aged children.	2006 and 2012	Sri Lanka and Kenya	[39,40]
Lot quality assurance sampling: A minimalistic approach of sampling originally designed for manufacturing inspection. It entails the collection and assessment of a minimal number of samples and the addition of more samples until statistical significance has been reached.	Not yet implemented	Not applicable	[34]
Pooling of samples to estimate STH prevalence while minimizing the number of diagnostic procedures: 50 School-aged children of 5 schools in each district were sampled. Individual examination was compared to pooled examination (pool size = 10) and both were processed with the Kato-Katz technique.	2017	Ethiopia	[41,42]

^1^ Still ongoing.

**Table 3:** Summary of records describing monitoring and surveillance systems for *Echinococcus* spp.

*Echinococcus* Species	Monitoring and Surveillance System	Time	Country	Reference
*E. granulosis*	Routine post-mortem inspection of slaughter sheep followed by mandatory sampling and cestoidal drug treatment of shepherd dogs at the farm of origin (in case of a positive result).	2007–2013	Tasmania	[44]
*E. multilocularis*	Annual surveillance system that includes fox sampling (300 annually) in each municipality of the country. In case of positive detection, intensification of fox sampling (to 3000 annually), collection of rodents and fox feces for analyzation, and intensification of national Norwegian surveillance system.	2000–2011	Sweden, Norway	[45,46,47,48]
*E. multilocularis*	Necropsy and coprological examination in addition to preventive deworming of free roaming, stray, and companion dogs.	2013–2017	Japan	[49]

**Table 4:** Summary of records describing monitoring and surveillance systems for *Taenia* spp.

*Taenia* spp.	Monitoring & Surveillance System	Time	Country/Region	Reference
*T. solium*	Observatory of Taeniasis and Cysticercosis: System consisting of several core activities mainly focusing on the detection and treatment of tapeworm carriers.	2017–…^1^	Portugal	[53]
*T. solium*	The integration of multiplex bead assays for parasitic diseases into national multiple indicator surveys.	2012	Cambodia	[54]
*T. solium*	Ring screening and treatment system: Pig surveillance by tongue palpation. When positive, test every individual living in a 100 meter radius for taeniasis and treatment with niclosamide.	2012–2014	Northern Peru	[55]
*T. saginata*	Conventional, routine meat inspection of bovines over 6 weeks of age: Visual inspection and incision of predilection sites.	1964–…^1^	Europe	[56,57,58]
*T. saginata*	Risk classification of farms/cattle and slaughterhouses based on data from history cases, serological tests, and other known risk factors. Subsequently, visual inspection of low-risk cattle and rigorous meat inspection of high-risk cattle.	Not yet implemented	United Kingdom, France, and Europe	[59,60,61]

^1^ Still ongoing.

**Table 5:** Summary of records describing integrated monitoring and surveillance systems at the control/elimination stage of *Taenia* spp., *Echinococcus* spp., *Schistosoma* spp., and soil-transmitted helminths (STHs).

Parasite Species	Monitoring & Surveillance System	Time	Country/Region	Reference
*Taenia saginata* and *Echinococcus granulosus*	A retrospective approach of spatial analysis for the integrated surveillance of both bovine cysticercosis and cystic echinococcosis combined with further actions based on the outcome.	2006–2010	Veneto (northeastern Italy)	[62]
*Schistosoma* spp. and STHs	Integrated cross-sectional surveys with anamnestic questionnaire for monitoring STHs and schistosomiasis.	2010	Taboo (south-central Côte d’Ivoire)	[63]
*Schistosoma* spp. and STHs	Screening for STHs, schistosomiasis, and polio using the global polio laboratory network.	2016–2017	Ghana	[64]

## Supplementary Materials

The following are available online at https://www.mdpi.com/2076-0817/9/1/47/s1, Checklist S1: PRISMA statement.
